# Case of Traumatic Tetanus

**Published:** 1864-05

**Authors:** L. Bartlett

**Affiliations:** New Bedford


CASE OF TRAUMATIC TETANUS, *Treated by Dr. L. Bartlett, of New Bedford. Apparent Relief from Strychnine.* [*Read by Dr. Henry I. Bowditch, at the meeting of the Suffolk District Medical Society, March 26th,* 1864, *and communicated by him to the Boston Medical and Surgical Journal.*]

Ezra Shaw, of Carver, Mass., called on me, Jan. 14th, in New Bedford, and gave the following account of himself. "Age 48 years; hight, five feet, eight inches; weight 135 lbs.; light hair and complexion, occupation farmer." Three weeks previously, while chopping in the woods, inflicted a pretty severe cut with his axe in the instep of the left foot. One week afterwards, while chopping in the woods (about Jan. 1st), cut off the thumb of the left hand, just below the second joint.

The foot soon healed, and the thumb was healing well up to the 7th and 8th of January, when he made two or three applications to it of "*burnt alum*, on account of proud flesh." The alum caused much irritation. On the 12th, began to experience difficulty in opening his jaws and in swallowing, and arranged to start for New Bedford. On the 13th, could only partially open his jaws; had pain in attempting to chew solid food, and considerable difficulty in swallowing. Started for New Bedford. Experienced some vexations and threatened delays on the way, which aggravated above symptoms.

When I first saw him, on the 14th, at 12 M., he could open his mouth about one third of the way, so as to protrude the tip of the tongue. He had but three teeth in the upper jaw, one incisor and one bicuspid on the right side, and one molar on the left side. He had frequent, almost constant chills, accompanied with sweating. Tongue coated; no appetite; complained of much pain through the jaws on attempting to open them or to swallow. Some pain in the back, and pain and stiffness in the legs, especially in walking. Some pain and stiffness in the muscles of the neck. Bowels constipated. Ordered hot foot-bath, to be continued for *one hour;* two compound cathartic pills at bed-time, to be followed by Nichols' cold infusion of senna in the morning. Flaxseed and meal poultice, well covered with *snuff* upon its surface, to be kept applied to the throat and sides of the neck. Flax-seed poultice to thumb.

15th, evening—Was somewhat relieved after foot bath, but passed a restless and distressed night. Cathartic operated in the morning. Had had frequent spasms of the jaws, biting his tongue—spasms of the muscles of deglutition on attempting to swallow. Pain through the jaws and throat constant and greatly aggravated by attempts to swallow. Jaws more closed and rigid. Muscles of back of neck rather stiff. Some spasmodic action of the diaphragm and muscles of abdomen during the night; chills and sweating have continued almost constantly since yesterday. Any movement of the body excited spasmodic action of the diaphragm and abdominal muscles ("drawing spells," he called them). Abdominal muscles tense and rather unyielding. No appetite. The tip of tongue only could be seen. Ordered ginger tea; beef tea; milk porridge. Foot-bath and poultice to be repeated. Five drops of strychnine in water every two hours (Marshall Hall's solution—one grain to the ounce).

16th—Symptoms much the same, but increased in severity. Legs somewhat relieved by foot-bath. Jaws more closed. Complained much of distress in the stomach, but said the medicine (strychnine) "always made it feel better." Ordered strychnine increased to eight drops every two hours. Teaspoonful tr. sumbul. every two hours in intervals of strychnine, for the distress in stomach. His general condition and symptoms grew gradually worse until the 19th. Omitted the sumbul after two doses, as "it distressed him." Had taken fifty drops of McMunn's elixir of opium on nights of 17th and 18th, but it nauseated and distressed him, as he said opiates always did. Bowels had been moved by cathartic medicine, and strychnine had been increased to ten drops every two hours, which, he said, "always made him feel better."

On the 20th, his jaws were firmly closed, and the spasmodic action of the muscles of the throat, diaphragm and abdomen were frequent and severe. The recti muscles of the abdomen seemed as rigid and tense as a ship's hawser, and perfectly unyielding to pressure. Had some spasms in his legs; muscles of neck stiff, neck bent a little backwards. Chills and sweating continued. Was able to swallow liquids, but with difficulty. Strychnine was now increased to fifteen drops every two hours—equal to three-eighths of a grain in twenty-four hours. Snuff poultice continued to throat, and bottles of hot water kept applied to back and legs. This treatment was continued until the 24th, during which time his symptoms became gradually ameliorated; took more nourishment, and could open his jaws a very little way; recti muscles less tense. He now showed some trembling spasmodic action in the legs and arms, which was attributed to the strychnine. He complained also that the medicine *now* distressed him. The strychnine was now omitted for twenty-four hours, and one grain of quinine every two hours was substituted.

He had no chills for two or three days, and the sweating was much diminished. Snuff-poultice was now omitted—the skin of the neck having become much irritated by it, and a bat of sheep's wool substituted for it.

On the 25th, continued to improve in all respects. Quinine was continued every four hours, and fifteen drops of strychnine in the intervals of the quinine, every four hours, up to 28th, when the quinine was omitted and the strychnine given only three times a day, fifteen drops at a time.

He could now open his mouth to nearly its full extent. The rigid tension of the recti muscles had passed off, but he had, occasionally, the "drawing spells" during the night.

Feb. 15th.—Has been steadily recovering since last date. Can open his mouth to full extent, and seems in all respects well. He has taken no medicine for a week, nor shown any symptoms of the disease. His thumb is not quite healed as yet.

He had no knowledge of the nature of his disease until after he became convalescent. Leaves for home this afternoon.

Marshall Hall's solution of strychnine: ℞ Acet. strychnia, gr. i; acet. acid, ʒss; alcohol rect., ʒij.; aq. dist., ʒvj. M. Dose five to twenty drops.

